# The moderator role of emotion regulation ability in the link between stress and well-being

**DOI:** 10.3389/fpsyg.2015.01632

**Published:** 2015-10-27

**Authors:** Natalio Extremera, Lourdes Rey

**Affiliations:** ^1^Department of Social Psychology, University of MalagaMalaga, Spain; ^2^Department of Personality, Evaluation and Psychological Treatment, University of MalagaMalaga, Spain

**Keywords:** emotion regulation, stress, gender differences, moderation, happiness, depression, emotional intelligence

## Abstract

This article examined the moderating role of a central core dimension of emotional intelligence—emotion-regulation ability—in the relationship between perceived stress and indicators of well-being (depression and subjective happiness) in a sample from a community adult population. The relationships for males and females on these dimensions were also compared. Results revealed that emotion-regulation abilities moderated both the association between perceived stress and depression/happiness for the total sample. However, a gender-specific analysis showed that the moderation effect was only significant for males. In short, when males reported a high level of perceived stress, those with high scores in regulating emotions reported higher scores in subjective happiness and lower depression symptoms than those with low regulating emotions. However, no interaction effect of regulating emotions and stress for predicting subjective happiness and depression was found for females. In developing stress management programmes for reducing depression and increasing well-being, these findings suggest that training in emotional regulation may be more beneficial for males than females. Our findings are discussed in terms of the need for future research to understand the different gender associations and to consider these differences in further intervention programmes.

## Introduction

Reviews of the general stress and coping literature have widely shown the role of stress as a contributor to important aspects of physical, cognitive and emotional maladjustment (Cohen et al., 1995, 2007). In addition, a significant relationship between stress and positive well-being indicators seems to exist. Therefore, when stress is not handled appropriately, it may have considerable influence on the development of negative emotional responses that can lead to reduced levels of well-being (Schiffrin and Nelson, 2010). Such negative consequences, however, might be avoidable and it is important to examine the personal resources that might have a buffering effect on the deleterious effects of stress, identifying potential targets for mental health interventions or well-being prevention programmes (Folkman, 2011).

### Emotion regulation ability and psychological functioning

Research suggests that the ability to regulate emotions is one potential key dimension that might reduce stress symptoms (Salovey et al., 1999; Sapolsky, 2007). Two relatively independent research traditions have developed that address emotion management (Peña-Sarrionandia et al., 2015). The first is the emotion regulation tradition, which focuses on the processes which permit individuals to influence which emotions they have, when they have them, and how they experience and express these emotions. The second is the emotional intelligence (EI) tradition, which focuses on individual differences in emotion abilities rather than on basic processes. It argues that the various instances of emotion regulation are not completely independent of one another within a given individual. The EI approach aims to provide a scientific approach for studying individual differences with regard to how people identify, use, understand and regulate their own emotions and those of others (Mayer and Salovey, 1997). We use the EI framework proposed by Mayer and Salovey (1997) for understanding how individual differences might influence perception, understanding and regulation of emotions in a relatively consistent manner which may explain why some people generally experience negative and positive emotions more frequently or intensely than others. Among the four core EI abilities proposed by Mayer and Salovey (1997), emotion-regulation ability (ERA), defined as the capacity to regulate one's own and others' emotional states, is consistently the most important facet associated with positive and negative psychological outcomes (Wranik et al., 2007; Côté et al., 2011; Ivcevic and Brackett, 2014). These emotional abilities are important for the prediction of psychological well-being indicators because individuals with greater ERA are thought to have a larger repertoire of strategies for maintaining desirable emotions and for reducing negative emotions (Mayer and Salovey, 1997). However, the way in which the ability to manage emotions affects coping with stress has been theorized in different ways (Salovey et al., 1999; Zeidner et al., 2006). A main effect hypothesis suggests that, independent of the amount of perceived stress experienced, high managing emotional individuals would have richer psychological resources, specifically for selecting and employing strategies when managing stressful situations and, therefore, improving interpersonal functioning and psychological well-being (Salovey et al., 1999). Accordingly, a great deal of research in the EI field has focused on the direct relation between EI and several mental health and social functioning outcomes (for a meta-analytic review, see Martins et al., 2010). With respect to specific relations of ERA, research has found that individuals with high ERA report greater well-being (Côté et al., 2010), better quality social interactions (Lopes et al., 2005) and lower motivation for revenge after a transgression (Rey and Extremera, 2014). In sum, empirical research has consistently shown that ERA generally contributes to better mental health and well-being relevant outcomes (Mayer et al., 2008).

### ERA and stress buffering effects

However, another possibility might be that ERA would have a stress-reducing effect by buffering the negative consequences of the stressful event (Salovey et al., 1999; Wranik et al., 2007). Thus, the impact of stress on positive and negative psychological outcomes is likely to be less profound when individuals possess higher abilities for managing emotions associated with stressful situations (Lazarus, 1999). Likewise, ERA may serve as a coping resource to reduce negative reactions when confronted with a stressful event (Salovey et al., 1999). Examining whether EI moderates the relationship between stressful events and mental and physical health indicators has been mostly studied with self-report EI measures (Ciarrochi et al., 2002); however, there is some preliminary evidence that supports how ERA using performance measures might reduce the negative impact of perceived stress in combination with some levels of emotional self-efficacy and levels of perceived stress (Gohm et al., 2005).

### ERA and gender differences

There appear to be consistent differences between men and women regarding emotional abilities, at least when assessed with EI performance measures (Brackett et al., 2004; Kafetsios, 2004). Thus, gender may differently influence the associations between emotional abilities and some negative mental health outcomes. For example, one study examining undergraduates found that high EI measured with performance measures was associated with reduced disruptive behavior in men, but not in women (Brackett et al., 2004). Similarly, low ERA was negatively associated with primary and secondary facets of psychopathy but only in undergraduate male students (Lishner et al., 2011). A more recent study also found high ability EI scores to be significantly associated with lower scores in depression in high school and college student men, but not in women (Salguero et al., 2012). It is unclear why ERA predicted higher mental health outcomes only for men; however, as Brackett et al. (2006) argued, although women are more skilled in the emotions domain than men, it is possible that emotional abilities play a different role in the psychological adjustment of men and women to the extent that the genders occupy different emotional worlds. Also, the ability to regulate emotions in females may be more internally determined and depend on a number of simultaneous factors, such as emotional states, more gender-specific differences in coping mechanisms (i.e., rumination) and the interpersonal context in which emotions are regulated (Salguero et al., 2015). In sum, these findings suggest that emotional qualities might play a differential role in negative psychological well-being outcomes, being especially stronger in males. Moreover, there is a well-established gender difference in the rates of depressive symptoms across most of the life span, with females showing more depression and stress (Matud, 2004) than males (Nolen-Hoeksema, 2001). On the contrary, some evidence suggests that women report experiencing greater happiness and positive mood (Diener et al., 1985). Gender differences in the use of emotion regulation abilities may explain, to some extent, sex differences in the prevalence of mood disorders or the promotion of well-being (Nolen-Hoeksema and Rusting, 2003; Thayer et al., 2003).

Beyond an examination of how emotional abilities may be associated with psychological adjustment, it is important to note that most studies on emotional abilities and gender differences have focused almost exclusively on negative psychological conditions (e.g., disruptive behavior, psychopathology, depression). Since the emergence of positive psychology (Seligman and Csikszentmihalyi, 2000), it has however become accepted that the absence of psychological maladjustment does not equate to the presence of positive functioning (Maddux et al., 2004). Therefore, low scores on measures that indicate the absence of depressive states do not indicate the presence of positive mood states. Rather, understanding of the psychological health requires consideration of the potential for people to also manifest psychological responses associated with enhanced psychological adjustment and well-being. Rather, elements of both negative (depression) and positive (well-being) adjustment can be experienced concurrently with different antecedents and mechanisms involved (Fava and Ruini, 2003). Accordingly, it is unclear how ERA relates to important positive psychological conditions, such as subjective happiness, and if the same gender specific findings with emotional abilities would be found.

### The present study

This study attempts to extend our understanding of the links between ERA, stress and positive and negative psychological outcomes in three ways. Firstly, we examine the relationship using a community adult population. One typical limitation of previous studies on EI, gender and psychological outcomes has been that most research has relied predominantly on college students. Compared with the general population, college students are likely to have stronger cognitive skills, less-well-formulated or crystalized attitudes and self-concepts, between others. Therefore, research with this subject population might exaggerate the magnitude of effects of situational influences and cognitive-emotional process on behaviors, raising issues of external validity (Reis and Judd, 2014). Secondly, the present study extends previous work by examining gender-based differences in the relationship between perceived stress and well-being outcomes and the relative role of ERA that may promote psychological outcomes differing by sex. Thirdly, while the majority of previous research has concentrated on the relationship between emotional abilities and well-being outcomes, the present study examines both the stress-reducing effect of ERA on the negative aspects of well-being (depression) as well as the positive (happiness) aspects.

At present, gender research on EI and ERA has been limited to critical issues related to negative conditions and psychopathology. Studies examining specific gender differences in the relationships between ERA and positive functioning have not been performed yet. Consequently, nothing is known about the extent to which the relationship between ERA and negative functioning is specifically moderated by gender or whether it reflects the same pattern with positive functioning as well. Gaining more insight into the specific gender pattern between ERA and positive and negative adjustment may help to improve the understanding of differential diagnosis and might have relevant implications for the manner and methods of conducting prevention and treatment strategies.

Therefore, given the possibility that the stress-reducing effect of ERA would be differently related to psychological outcomes in males and females, we conducted gender-specific regression analyses. Since the strongest and most reliable finding was that males are typically found to take better advantage of having emotional abilities than females, we expected that the predictive relationship between ERA, both alone and when interacting with stress, would be more significant for men than for women.

## Method

### Participants and procedure

A convenience sampling method was used to collect data in 2012 from Spanish speaking adults who volunteered to take part in the study. Participants received a paper-and-pencil questionnaire from our research assistant. Altogether, 677 surveys were received. Participants were requested to voluntarily answer all the questions in private, and to return the completed questionnaire. Each respondent completed a questionnaire containing a letter, in which the goal of the study was briefly introduced, and the confidentiality and anonymity of the answers were underlined. In this letter, socio-demographic data, such as age, sex, and occupations, were also requested. Twelve participants were excluded from further analyses because they failed to complete all instruments. Hence, the responses provided by the remaining 665 participants were used (336 females and 329 males) with mean age = 35.69 years (standard deviation = 11.99 years, age range = 18–68 years). Over 50% of the respondents were single and 65.4 were employees working in a wide range of sectors. These included manufacturing, health services, human services, public administration, and education. This study was carried out in accordance with the Declaration of Helsinki and ethical guidelines and approved by the Research Ethics Committee at the University of Málaga.

### Materials

*Perceived stress* was measured by the Perceived Stress Scale (PSS; Cohen et al., 1983), which is a 14-item measure of self-appraised stress (e.g., “*In the last month how often have you been upset because of something that happened unexpectedly?*”). Respondents are asked to rate the frequency of items across a five-point Likert-type scale ranging from 0 (never) to 4 (very often). Respondents are asked to rate the frequency of experiencing stress during the last month. The shorter 4-item version of the PSS was used in the present study. We used a well-validated Spanish version (Remor and Carrobles, 2001). Cronbach's alpha in this study was 0.70.

*Emotion-Regulation ability* was measured using a situational judgment test: the managing emotions section of the Mayer-Salovey-Caruso Emotional Intelligence Test (MSCEIT Version 2.0; Mayer et al., 2003). This subscale is measured with two tasks: an emotion management task (five parcels; four responses each) and an emotional relationships task (three item parcels; three responses each). In the former, respondents are asked to judge the actions that are most effective in obtaining the specified emotional outcome for an individual in a story. In the latter, respondents judge the actions that are most effective for one person to use in the management of another person's feelings in social situations. There are a total of 29 items assessing managing emotions. MSCEIT v.2.0's psychometric properties were appropriate and convergent and discriminant validity was successfully demonstrated (Mayer et al., 2003). We used a well-validated Spanish version (Extremera et al., 2006). Split-half reliability for the Spanish emotion regulation subscale of the MSCEIT in this study was 0.85.

*Well-being outcomes* were measured by the Subjective Happiness Scale (SHS; Lyubomirsky and Lepper, 1999) and the depression subscale from the 21-item Depression, Anxiety and Stress Scale (DASS-21; Lovibond and Lovibond, 1995). The SHS is a four-item measurement of global subjective happiness. Two items ask respondents to describe themselves using both absolute ratings and ratings relative to peers, while the other two items offer brief descriptions of happy and unhappy individuals and ask respondents about the extent to which each description describes them. Each item was assessed on a 7-point Likert scale (e.g., “In general I consider myself: 1 = *Not a very happy person* to 7 = *A very happy person”*). The SHS has shown high internal consistency, high test–retest and self-peer correlations reliability and high convergent and discriminant validity. We used a well-validated Spanish version (Extremera and Fernández-Berrocal, 2014). Cronbach's alpha in this study was 0.73. The depression subscale from DASS-21 consists of seven item with a Likert-type scale, designed to measure the negative emotional states of depression in the past week, with “0 = did not apply to me at all” to “3 = applied to me very much, or most of the time” (e.g., “*I couldn' t seem to experience any positive feelings at all*”). The Spanish version showed satisfactory internal consistency, and adequate divergent and convergent validity (Bados et al., 2005). Cronbach's alpha in this study for the three subscales ranged from 0.85 to 0.89.

## Results

### Gender differences on ERA and positive and negative psychological outcomes

Univariate differences were tested using one-way Analysis of Variance (ANOVA). To provide an estimate of the magnitude of differences by sex, we calculated the effect size, reported as Cohen (1988). The results are presented in Table 1. With respect to ERA, females scored significantly higher with a small effect size. In our study, no significant differences were found for perceived stress, depression and subjective happiness between females and males.

**Table 1:** **Gender differences in study variables**.

	**Total sample *N* = 665 *M* (*SD*)**	**Female *N* = 336 *M* (*SD*)**	**Male *N* = 329 *M* (*SD*)**	***p***	***d***
Perceived stress	1.37 (0.68)	1.40(0.65)	1.33(0.70)	0.20	–
Emotion regulation ability	100 (14.35)	101.57(14.19)	98.40(14.36)	0.01	−0.26
Depression	7.34 (8.48)	7.68(8.72)	6.99(8.23)	0.29	–
Subjective happiness	5.23 (0.96)	5.29(0.94)	5.18(0.97)	0.14	–

### Descriptive analyses

Bivariate correlations among study measures are displayed in Table 2, presented separately for females and males. As shown in the table, perceived stress was positively and significantly related to depression symptoms and negatively associated with ERA and subjective happiness scores for both females and males. Similarly, ERA was negatively and significantly related to depression symptoms and positively associated with subjective happiness for both genders.

**Table 2:** **Intercorrelations among measures separately for females and males**.

	**1**	**2**	**3**	**4**
Perceived stress (PSS)	–	−0.27^**^	0.58^**^	−0.51^**^
Managing emotions (MSCEIT)	−0.34^**^	–	−0.34^**^	0.18^**^
Depression (DASS)	0.60^**^	−0.42^**^	–	−0.49^**^
Subjective happiness (SHS)	−0.58^**^	0.35^**^	−0.51^**^	–

*N = 665*;

** *p < 0.01*.

*Coefficients above the diagonal are for females (N = 336), those below the diagonal are for males (N = 329)*.

### Hierarchical regression analyses

Finally, to test for a potential moderating effect of ERA in stress-psychological outcomes, several separate analyses were conducted with depression and happiness as the dependent variables for the total sample, both females and males. In the first step, age was entered as a covariate. Scores for perceived stress were entered in Step 2. In step 3, ERA scores were entered. Finally, the interactions between stress and ERA following centring procedures were entered in the third step to explore the moderating effects (Aiken and West, 1991). The results of these analyses are presented in Table 3.

**Table 3:** **Regression results for the moderating effect of perceived stress and ERA on depression and happiness for total, female and male sample**.

		**Total sample**			**Female sample**			**Male sample**		
		**β**	***R*^2^**	**Δ*R*^2^**	**β**	***R*^2^**	**Δ*R*^2^**	**β**	***R*^2^**	**Δ*R*^2^**
	Depression		0.39			0.37			0.42	
Step 1										
	Age	0.02		0.00	−0.18		0.00	0.06		0.00
Step 2										
	Perceived stress	0.53^**^		0.35^**^	0.54^**^		0.33^**^	0.52^**^		0.36^**^
Step 3										
	ERA	−0.18^**^		0.04^**^	−0.17^**^		0.03^**^	−0.22^**^		0.05^**^
Step4										
	Stress X ERA	−0.07^*^		0.005^*^	0.03		0.004	−0.10^*^		0.01^*^
	Subjective happiness		0.34			0.29			0.40	
Step 1										
	Age	−0.14		0.00	−0.13		0.011	−0.14		0.00
Step 2										
	Perceived stress	−0.53^**^		0.32^**^	−0.52^**^		0.27^**^	−0.54^**^		0.36^**^
Step 3										
	ERA	0.09^*^		0.01^**^	0.01		0.003	0.15^**^		0.03^**^
Step4										
	Stress X ERA	0.09^**^		0.01^**^	0.09		0.006	0.10^*^		0.01^*^

* *p < 0.05*;

** *p < 0.01*.

As can be seen in Table 3, we found that the stress X ERA interaction model was significant for depression and subjective happiness for the total sample. When we further examined these moderating models for females and males separately, we found that the interaction models were only significant for males but not for females.

As seen in Table 3, for females, perceived stress was a consistent predictor of depression and happiness (the explained variance ranged from 29 to 37%). ERA was a significant predictor for depression (β = −0.17; *p* < 0.01) in females. However, ERA was not a significant predictor of subjective happiness. Similarly, we found no interaction effects between stress and ERA which did not explain positive and negative well-being outcomes in females.

For males, as expected, perceived stress was a significant predictor of depression and subjective happiness (36% explained variance in both cases). Thus, in Step 3 we found a significant main effect of ERA in predicting both negative and positive well-being outcomes beyond perceived stress levels (the explained variance ranged from 3 to 5%). Finally, in the last step, we found a significant increase in variance for the interaction effect for both depression and subjective happiness. In short, only for males, the stress x ERA interaction explained a small, unique and significant portion of variance in depression and subjective happiness (Δ*R*^2^ = 0.01), beyond the variance contributed by significant main effects for both perceived stress and ERA.

**Figure 1:**
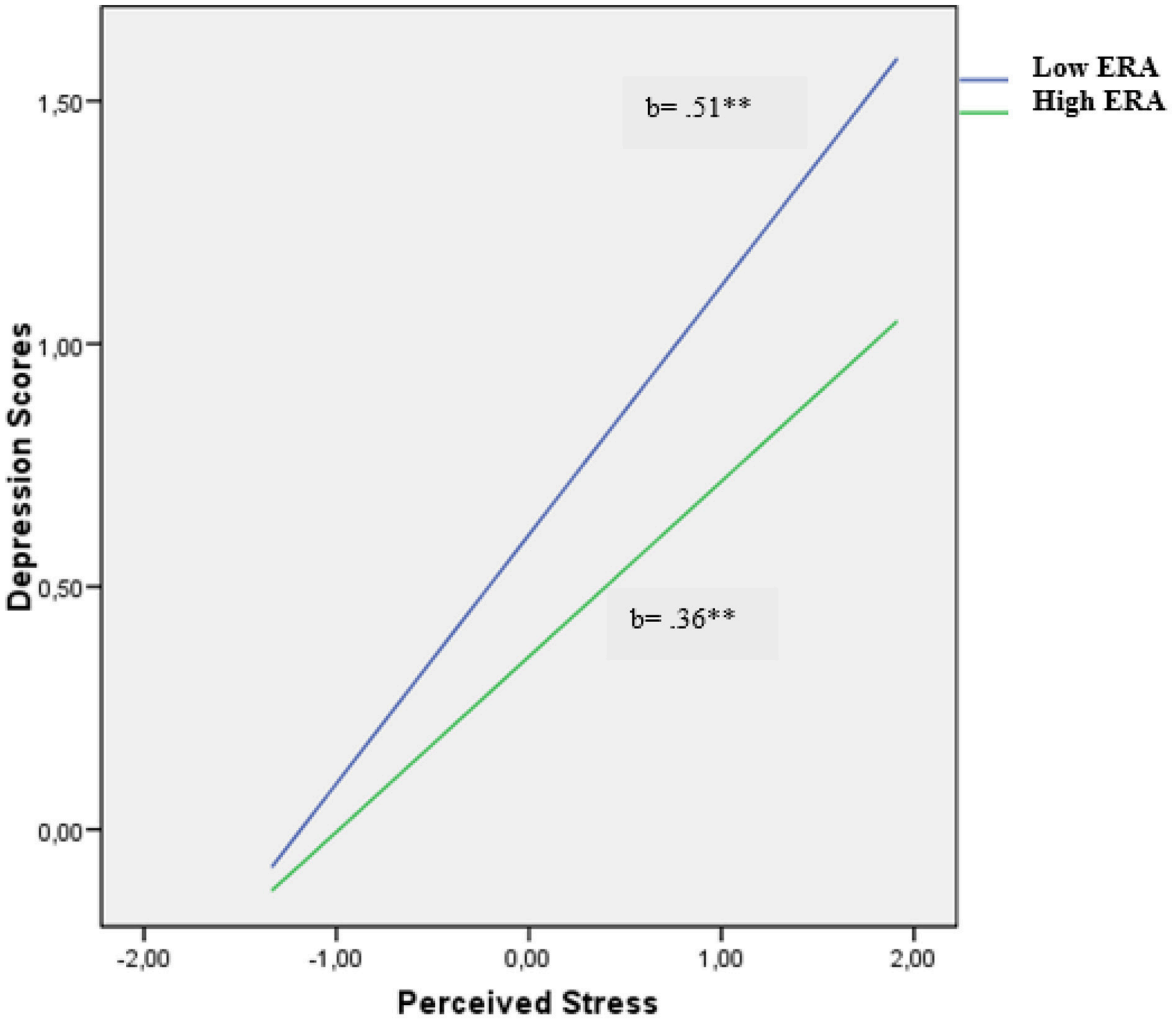
**Relationship of perceived stress and emotion regulation ability for predicting depression scores in males**. ^**^p < 0.01.

To illustrate the perceived stress x ERA interaction for depression and subjective happiness in males, we plotted the regression following the procedures outlined by Hayes and Matthes (2009). For depression symptoms, as Figure 1 shows, the relationship between perceived stress and depressive symptoms weakened as levels of ERA increased. Yet, importantly, there was a significant positive relation between perceived stress and depression at high levels of ERA [*b* = 0.36, *t*_(329)_ = 7.49, *p* < 0.001]. Similarly, at low levels of ERA, the relationship between perceived stress and depression was also significant [*b* = 0.51, *t*_(329)_ = 9.77, *p* < 0.001].

**Figure 2:**
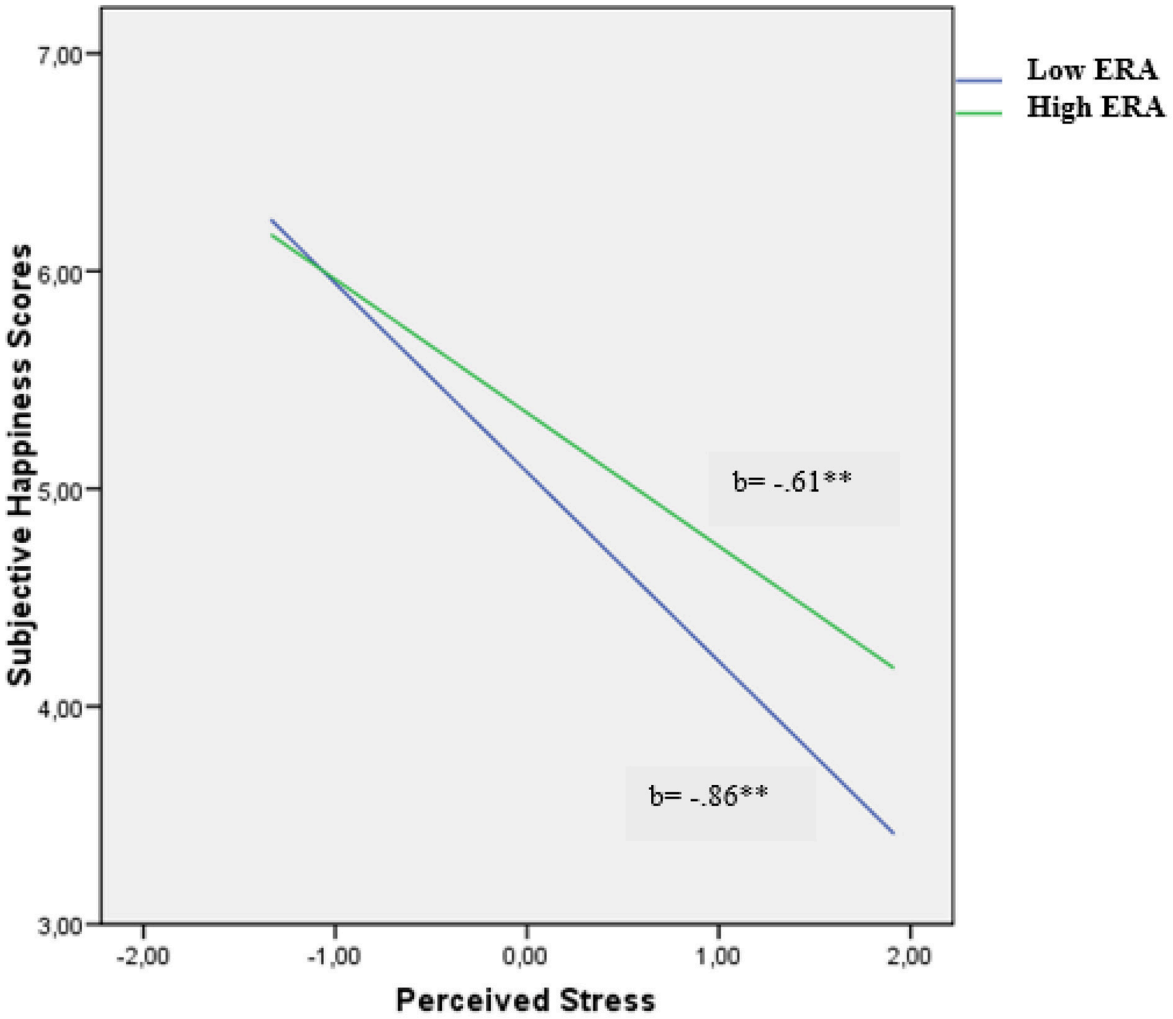
**Relationship of perceived stress and emotion regulation ability for predicting subjective happiness scores in males**. ^**^p < 0.01.

For subjective happiness, we found a similar pattern. As Figure 2 shows, the relationship between perceived stress and low subjective happiness weakened as levels of ERA increased. Yet, there was a significant negative relation between perceived stress and subjective happiness at high levels of ERA [*b* = −0.61, *t*_(329)_ = −7.35, *p* < 0.001]. Similarly, at low levels of ERA, the relationship between perceived stress and depression was also significant [*b* = −0.86, *t*_(329)_ = −9.57, *p* < 0.001].

## Discussion

Over the past decades, there has been a growing interest in the psychological correlates involved in stress–well-being outcomes. Recently, emotional abilities comprising the EI construct, and in particular ERA, have appeared as a key dimension in predicting both stress and different indicators of psychological adjustment and well-being (Zeidner et al., 2006; Mayer et al., 2008). Nevertheless, studies examining the potential moderating effect on the association between stress–well-being outcomes and ERA have been neglected. Using a community sample, this article examined the moderating role of ERA in the relationship between stress and both negative (depression) and positive well-being (happiness) outcomes. Thus, the potential moderating effect was compared for males and females.

With regard to the relationships between examined variables, perceived stress was positively and significantly related to depression symptoms and negatively associated with ERA and subjective happiness. Our findings were in line with our hypothesis that high scores on perceived stress may lead to increased levels of emotional disturbances and reduced levels of well-being and consistent with prior studies finding that people reporting higher stress experienced a much larger decrease in happiness and lower health indicators (Cohen et al., 2007; Schiffrin and Nelson, 2010). Similarly, ERA was negatively and significantly related to depression symptoms and positively associated with happiness. These findings were congruent with earlier work showing that ERA is associated with a lower tendency to experience psychological maladjustment (Hertel et al., 2009) and report greater well-being (Côté et al., 2011). Thus, individuals might handle challenging or threatening events successfully through an effective ability to regulate positive and negative emotions, both in self and other. ERA might minimize the impact of stress and alleviate its negative consequences, which should be a protective factor of psychological problems across the life course.

With respect to examining the gender differences in depression, happiness and stress, contrary to expectation, this study did not find statistically significant differences in females' levels of depressive symptoms, perceived stress and happiness, relative to males (although the mean levels were higher for women in all dimensions). It could be that the measure used to assess outcomes limited the amount of potential variability or it is possible that a larger sample is necessary for statistically significant gender-differences. Nevertheless, we found that ERA interacted differently as a function of gender specifically, extending the previous literature on gender differences on the influence of EI abilities in psychological outcomes (Brackett et al., 2006; Salguero et al., 2012). In short, some preliminary evidence was found suggesting significant differences in the associations between ERA and perceived stress-happiness and perceived stress-depression for males, but not for females. Specifically, when stress was high, males at the low ERA level showed higher depression and lower happiness. Further, our findings suggest that ERA might be more related to the stress–well-being outcomes in males, while it would be less relevant or would imply other psychosocial factors than ERA for females. It is possible that ability to regulate emotions in females may be more internally determined and depend on a number of simultaneous factors, such as emotional states, more gender-specific coping mechanisms (i.e., rumination) and the interpersonal context in which emotions are regulated. This suggests that there may be other important unmeasured moderators/mediators not included in this study that may be accounting for the specific gender effect between emotional abilities and psychological outcomes (Salguero et al., 2015).

One possible explanation for the moderating effect of ERA not being present for females concerns the possible existence of a threshold effect (Brackett et al., 2004). Pending empirical confirmation of this theoretical argument, there may be a minimum level of ERA that is needed to function effectively in everyday life, and the proportion of men who fall below this threshold may be higher than the proportion of women. Because women have higher ERA scores than men, women may have attained that threshold and men need a lower threshold of ERA than women to gain better subjective well-being. Differences in ERA scores for women, then, would not explain variance in psychological outcomes. Another explanation might be due to the quite different emotional worlds that men and women inhabit; therefore, emotional abilities might operate differently in men and women (Shields, 2002). Alternatively, it might be that men, as a group, do not take the test as seriously as women. In conclusion, these findings underline the need for gender-specific approaches to advance research on this subject and further research should conduct analyses separately by gender, when possible.

If replicated, the findings have implications for research and clinical practice. As a result, if ERA influences male and female tendencies to experience positive and negative well-being outcomes in distinctive ways, EI programmes focused on increasing well-being through development of emotional skills may not have a uniform effect on both females and males. Such research may ultimately suggest that different types of EI interventions are required for males and females. Our present data might imply that gender differences may be important when using cognitive-based therapies for reducing depression and increasing well-being. Intervention programmes that instruct patients in the use of ERA may benefit from our findings that males may not have as many emotional abilities available for reducing negative moods. Therapists can assist males in helping to identify and cope with the emotions produced by stressful events, understanding and evaluating their feelings, as well as increasing emotional abilities to modify them. Disrupting the spiraling cycle of negative emotions and increasing positive ones can be accomplished by identifying and developing activities and emotional regulation strategies to help the individual manage his/her feelings to increase positive emotions and reduce the biasing effect of negative mood on cognition. Alternatively, these findings might suggest that in an EI intervention, male patients would be more easily trained in ERA and have greater efficiency in increasing their positive emotional states than females.

Besides, many research study results consistently indicate that women score significantly higher in emotional disturbances than do men (Kessler et al., 1993; Alonso et al., 2004). However, our result support that women typically score higher than men in ERA, but it is possible that although having high ERA levels, possibly do not put them in action because they are not confident in these abilities, thus preventing them from using adequate strategies to manage the negative affect, which could eventually lead to increased depressive symptoms (Salguero et al., 2015). Therefore, this association between ERA and well-being might be dependent on EI self-perceptions for women. That is, women would report less negative affect (or high positive affect) only if they had higher levels of ERA and also tend to perceive themselves more skilled in their emotional abilities (Salguero et al., 2015). Alternatively, while healthy women were used in this research, different patterns might be found between healthy and clinical participants. For example, healthy women might use these emotional abilities, independent of levels of emotional self-efficacy, which might contribute to well-being. To fully understand these issues, further research should examine and confirm our results in clinical populations involving clinically depressed and non-depressed women, broadening our understanding about the whole spectrum of individual variation that explain emotional disturbances in women. Obviously, there are other factors than ERA that may influence increased vulnerability in women to mood disorders such as possible differences in specific emotion regulation strategies (Nolen-Hoeksema, 2012), underlying factors linked to cognitive/executive control (Channon and Green, 1999) or potential brain-personality mechanisms protecting against emotional disturbances (Llewellyn et al., 2013; Dolcos et al., 2015). Additional research should examine whether the relationship between stress and well-being indicators may be through other psychosocial mechanisms of deriving well-being, not only ERA, and also whether the form and quality of these relationships affect the psychological functioning of men and women differently.

When considering the implications of these findings, some limitations are certainly warranted. First, our study design was cross-sectional and our data may be bi-directionality. ERA, stress and well-being should be linked not only concurrently but over time, with further analysis of the lead-lag relationships. In addition, such relations should be tested at the within-person level rather than only in terms of individual differences, which would require a future longitudinal and experience-sampling design. Thus, the inclusion of new and improved measures of anxiety, stress and depression (i.e., a diagnostic interview, biomedical measures, clinician-administered instruments) would provide a comprehensive perspective and would generate some new insights into the role that gender might play in shaping the multiple health benefits of ERA. Similarly, our study was based on a convenience sample from a community population, not on a clinical sample. Our findings from this relatively non-distressed and healthy sample may not necessarily generalize to clinic or distressed-based samples. Finally, although ERA was found to be differently associated with positive and negative adjustment as a function of gender, it is important to bear in mind that other variables affecting stress and well-being facets (e.g., health, socioeconomic status or personality factor, among others) might be important predictors of psychological functioning and must be taken into account in further research.

Notwithstanding the above-mentioned limitations, there are important contributions in this study. This study suggests an empirical framework for testing ERA in gender-specific models as a moderator of the relationship between stress and negative and positive well-being outcomes. Given that results have been found in a normal adult population, it provides meaningful evidence for the external validity of ERA as a predictor of well-being outcomes in community-based samples. This study also provides preliminary evidence of the gender differences in the relationship between stress, ERA and well-being outcomes. Finally, the higher contribution of ERA to happiness and depression in males may help males develop more emotional regulation strategies, and this finding provides guidance on how to implement intervention programmes, especially in males, aimed at enhancing well-being and reducing psychosocial maladjustment.

### Conflict of interest statement

The authors declare that the research was conducted in the absence of any commercial or financial relationships that could be construed as a potential conflict of interest.
